# Study on the pattern of spermatogenesis during the breeding season of the Chinese soft-shelled turtle, *Pelodiscus sinensis* (Reptilia: Trionychidae)

**DOI:** 10.1530/RAF-22-0117

**Published:** 2023-03-23

**Authors:** Yu-Fei Yang, Jia-Hao Wu, Run-Lan Lin, Shang-Jun Yin, Guo-Ying Qian, Wei Wang, Yong-Doo Park

**Affiliations:** 1 College of Biological and Environmental Sciences, Zhejiang Wanli University, Ningbo, Zhejiang, PR China; 2 Ningbo Jiangshan High School, Ningbo, Zhejiang, PR China; 3 Skin Diseases Research Center, Yangtze Delta Region Institute of Tsinghua University, Jiaxing, Zhejiang, PR China; 4 Zhejiang Provincial Key Laboratory of Applied Enzymology, Yangtze Delta Region Institute of Tsinghua University, Jiaxing, Zhejiang, PR China

**Keywords:** spermatogenesis, sperm cell development, maturation, seasonal reproduction, pattern

## Abstract

The Chinese soft-shelled turtle, *Pelodiscus sinensis* (Reptilia: Trionychidae), is a typical seasonal breeding species, and its spermatogenesis pattern is complex. In this study, the process of sperm cell development was studied using histology. The process of sperm cell development may be divided into six stages based on a combination of different cell types in the seminiferous epithelium. A close examination revealed two patterns of sperm cell development in the seminiferous tubules during the breeding season. The first is a normal sperm cell development pattern, in which the process of sperm cell development and maturation are completed in the seminiferous epithelium without round spermatozoa in the lumen. The second is rapid sperm cell development, in which the first batches of round spermatozoa fall off the seminiferous epithelium before they mature, thus beginning a second batch of sperm cell development. The round sperm cells are shed into the lumen and further mature in the seminiferous tubules and epididymis. This rapid sperm cell development process of the Chinese soft-shelled turtle is rare in other vertebrate species and may be an adaptation to cope with seasonal breeding. The results of this study provide insight into the theory of seasonal reproduction in reptiles.

**Lay summary:**

The Chinese soft-shelled turtle is a typical seasonal breeding species, and the process of sperm cell development is complex. In this study, the process of sperm cell development is divided into six stages in Chinese soft-shelled turtles and revealed two patterns of sperm cell development in the testis during the breeding season. The first is a normal sperm cell development pattern, in which the process of sperm cell development and maturation are completed in the testis. The second is a rapid pattern, in which the round sperm cells fall off before they mature and further mature in the epididymis. This rapid sperm cell development process of the Chinese soft-shelled turtle is rare in other vertebrate species and may be an adaptation to cope with seasonal breeding. The results of this study provide insight into the theory of seasonal reproduction in reptiles.

## Introduction

The process of sperm cell development is complex and refers to the process of spermatogonial cells transforming into sperm over a long time within the boundary of seminiferous tubules of the testis. This process is primarily regulated by the interaction between the hypothalamic pituitary testicular axis, interstitial cells, and the brain (Cheng & Mruk 2002, Mruk & Cheng 2004, Hess & Franca 2008, Suede *et al.* 2022). Reptiles occupy a strategic position in the evolutionary history of vertebrates, but there are few studies on sperm cell development. The process of sperm cell development is similar in reptiles and mammals in general, and the main process includes included cell volume changes and cytoplasmic removal (Sprando & Russell 1988). For reptiles, the process of sperm cell development is more seasonal (Gribbins & *Gist* 2003, Gribbins *et al.* 2008). However, some reptiles can produce sperm continuously throughout the year (Gribbins *et al.* 2011). The aim of this study was to provide new data on the process of sperm cell development in the Chinese soft-shelled turtle.

The process of sperm cell development exhibits typical seasonal characteristics during the annual cycle in the male Chinese soft-shelled turtle. It initiates and progresses continuously in the month of April in the epithelium of the seminiferous tubules, reaches a peak during reproduction in August and September, and continues through November (Cooke & Saunders 2002, Zhang *et al.* 2007, Avidor-Reiss *et al.* 2020). To adapt to seasonal breeding behavior, spermatozoa may be stored in the epididymis of males and the genital tract of the females for a long time (Orr & Zuk 2014). Therefore, the sperm must be periodically released from the seminiferous epithelium. The ability to divide the cycle of the seminiferous epithelium into identifiable component stages is an important prerequisite for the study of the process of sperm cell development (Reddy & Prasad 1970, Berndtson 1977). This division is common in mammals. For example, based on the morphological characteristics of tubules and the development of the acrosome system, the bovine seminiferous epithelial cycle may be divided into 12 stages (Berndston & Desjardins 1974, Staub & Johnson 2018), whereas the donkey seminiferous epithelial cycle is divided into 8 and 12 stages to study the process of sperm cell development and the seminiferous epithelium more systematically. This contributes to an understanding of spermatogonial cells in the testis of the mule and spermatogonial transplantation in the two species (Neves *et al.* 2002, Chiarini-Garcia *et al.* 2009). In addition, 6, 12, and 14 specific stages were identified in human, mouse, and rat according to the morphology of germ cells at different developmental stages (Leblond & Clermont 1952, Oakberg 1957, Hermo *et al.* 2010). The combined classification scheme of these cells is essential for understanding the process of sperm cell development, because it provides a description and quantification of seminiferous epithelium to reveal the cycle of the seminiferous epithelium (Amann 2008).

In addition, studies have found that sperm stored in the epididymis of the Chinese soft-shelled turtle is heterogeneous including round and immature germ cells that appear in the lumen (Chen *et al.* 2015, Chen *et al.* 2020*a*) and are discharged from the testicular lumen. Currently, there is no research to describe this phenomenon, and, to our knowledge, quantitative information on the process of sperm cell development of the soft-shelled turtle, such as the detailed histology of the cycle of the seminiferous epithelium, has not been reported. The objectives of the present study were to document the process of sperm cell development model and the distinct cellular associations of the cycle of the seminiferous epithelium in the male Chinese soft-shelled turtle. This will provide insight into the process of sperm cell development in reptiles and improve their reproductive efficiency. It will also contribute new methods and strategies for improving the yield of the turtles.

## Materials and methods

### Animals

Ten healthy male soft-shelled turtles (mature 3–4 years old) were purchased from the Walmart supermarket food section in Ningbo city, Zhejiang Province, China, during autumn (September and October). Pentobarbital sodium (40 mg) was injected intraperitoneally to anesthetize the turtles over a period of 15 min. The animals were sacrificed, and the testicles and epididymal tissue were carefully excised and washed repeatedly with phosphate-buffered saline (PBS). Tissue from the head of the epididymis was also collected. A slight surface cut was made with a scalpel to allow the semen to flow out naturally. The operation was carried out under sterile conditions.

The ethical standards for the animal protocol passed an ethical review by the Experimental Animal Ethics Committee of Zhejiang Wanli University. The approval number for the ethical review is #2020070101.

### Histological methods

The excised testicular tissue (from September) was cut into 5 mm square blocks and fixed with 4% paraformaldehyde for 48 h. After alcohol dehydration, xylene clearing, and paraffin treatment, the paraffin-embedded tissues were sectioned at a thickness of 6 μm. After staining with hematoxylin and eosin (H&E), According to morphological characteristics of cytoarchitecture, spermatogenic cell types were judged (Zhang *et al.* 2008, Marín-Gual *et al.* 2022). The changes of spermatogenic cell assemblages in spermatogenic epithelium were compared in different seasons. The tissue sections were observed, and images were captured using a Leica-DM4000 microscope. Based on the analysis of the images, the AI (Adobe Illustrator) software was used for drawing.

For Alcian blue–periodic acid thickness (AB-PAS staining), the slices (September) were stained with 0.1% Alcian blue reagent for 10 min, washed with distilled water for 5 min, and stained with 0.5% periodic acid for 5 min after washing. Then, the slices were stained with Schiff reagent for 15 min, washed with distilled water, and stained with hematoxylin for 20 s. After dehydration and sealing, the slices were observed and photographed under an optical microscope.

For eBioscience Propidium Iodide (PI) staining, 50 μL semen were taken from the epididymal head. Then, 3 mL of PBS were added and mixed. Following centrifugation for 10 min, 5 mL of 70% ethanol were added and mixed well at 4℃ to avoid light overnight. The next day, the samples were centrifuged, washed with PBS to remove excess ethanol, and re-centrifuged to remove the supernatant. A precipitate was obtained after 3 mL of PBS werfe added. Next, 1 mL from the sample tube was divided into two tubes representing each month. After checking quality control, PI dye solution was added, mixed, and incubated in the dark for 15 min. The samples were analyzed by flow cytometry.

## Results

### Cycle of the seminiferous epithelium

**Figure 1:**
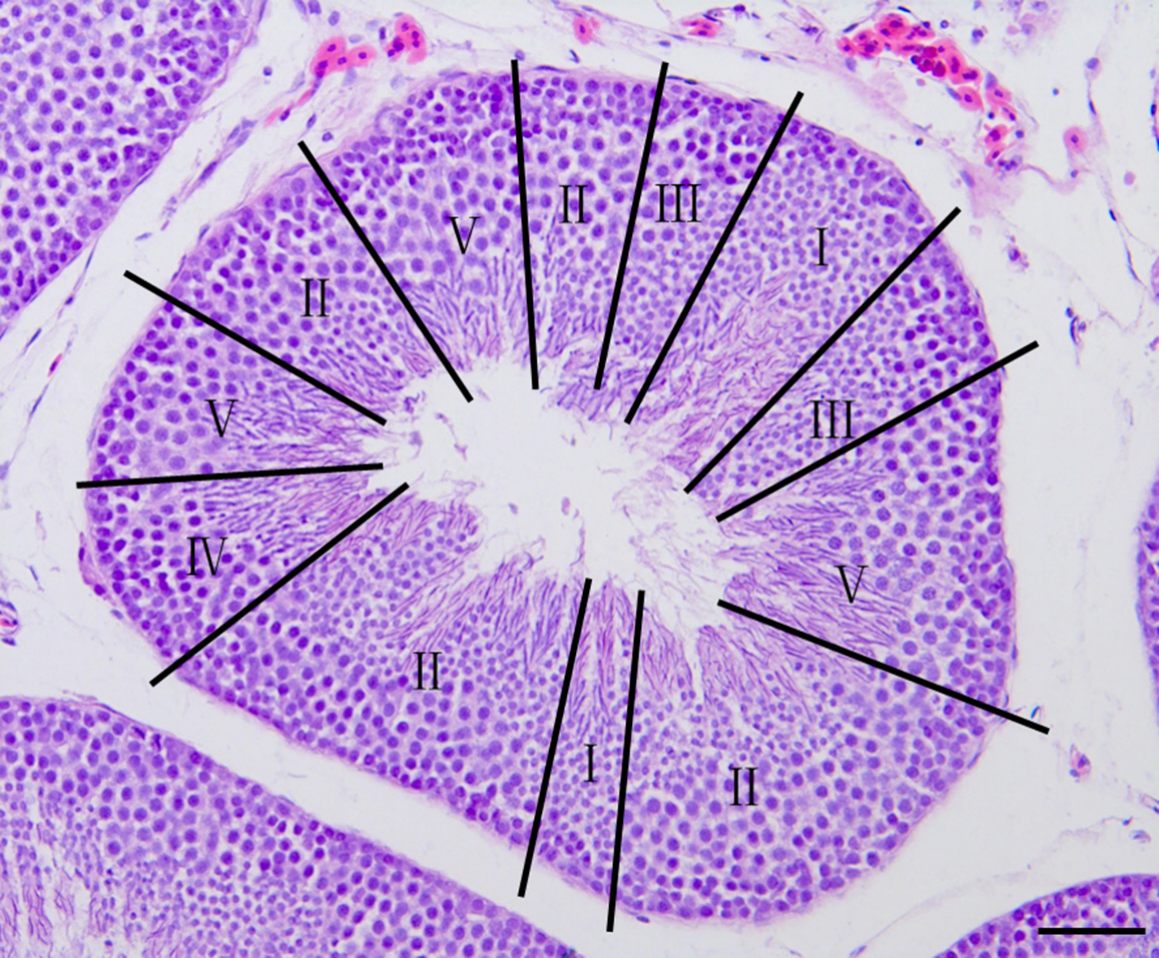
Photo under low magnification of tubular section shows the distribution of the various typical cell associations or stages of the cycle. They are marked with Roman numerals and rotate clockwise to form a ‘fan’ pattern. Staining: hematoxylin and eosin (scale bar: 100 μm).

**Figure 2:**
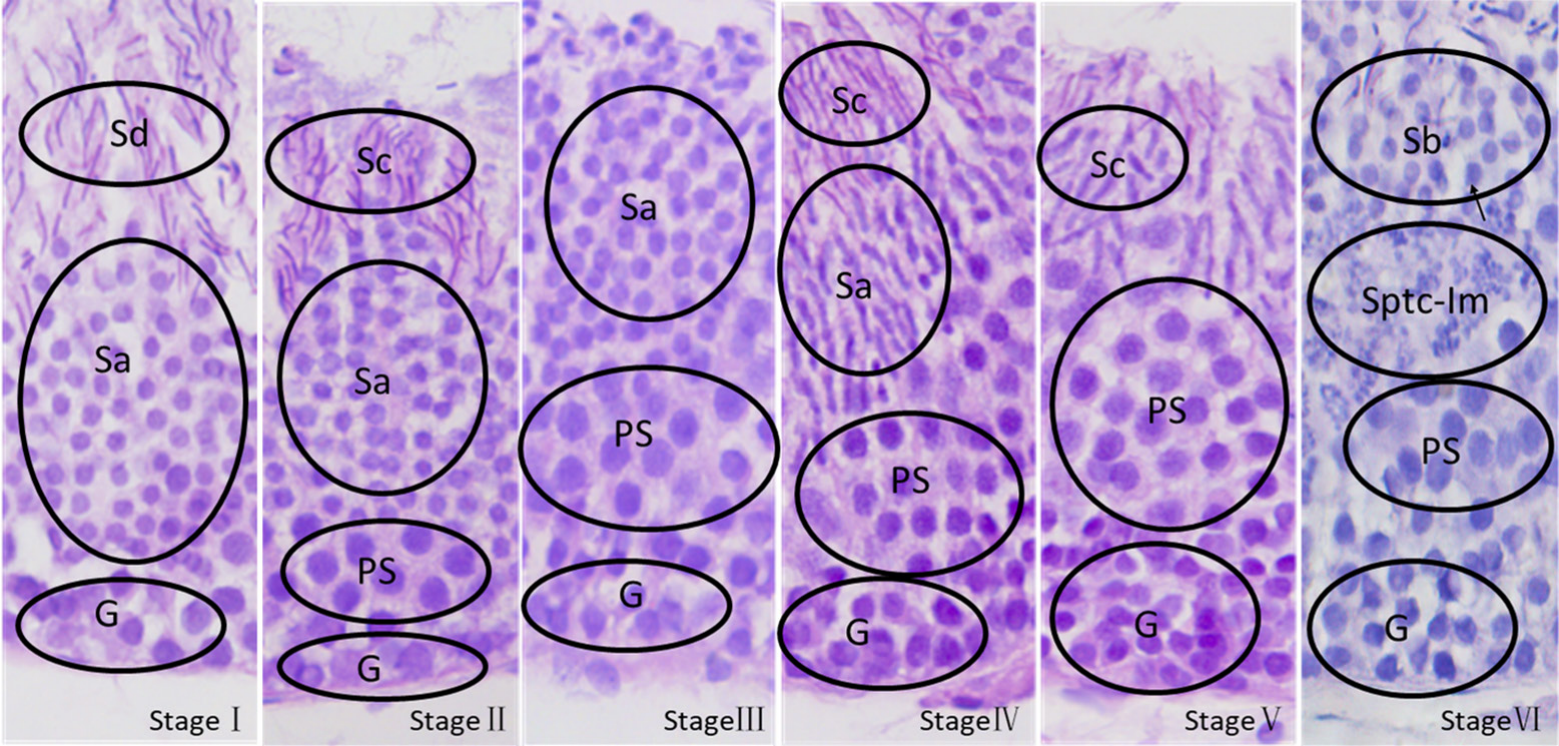
Microphotographs of areas of the soft-shelled turtle seminiferous epithelium show the six typical cellular associations corresponding to the six stages of the cycle of the seminiferous epithelium. The number of stages is marked with Roman numerals in each picture. G, spermatogonia; PS, primary spermatocytes; Sptc-Im, spermatocytes in division; Sa, Sb, sperm cell types of different generations; Sc, Sd, sperm of different generations; magnification 400 times.

**Figure 3:**
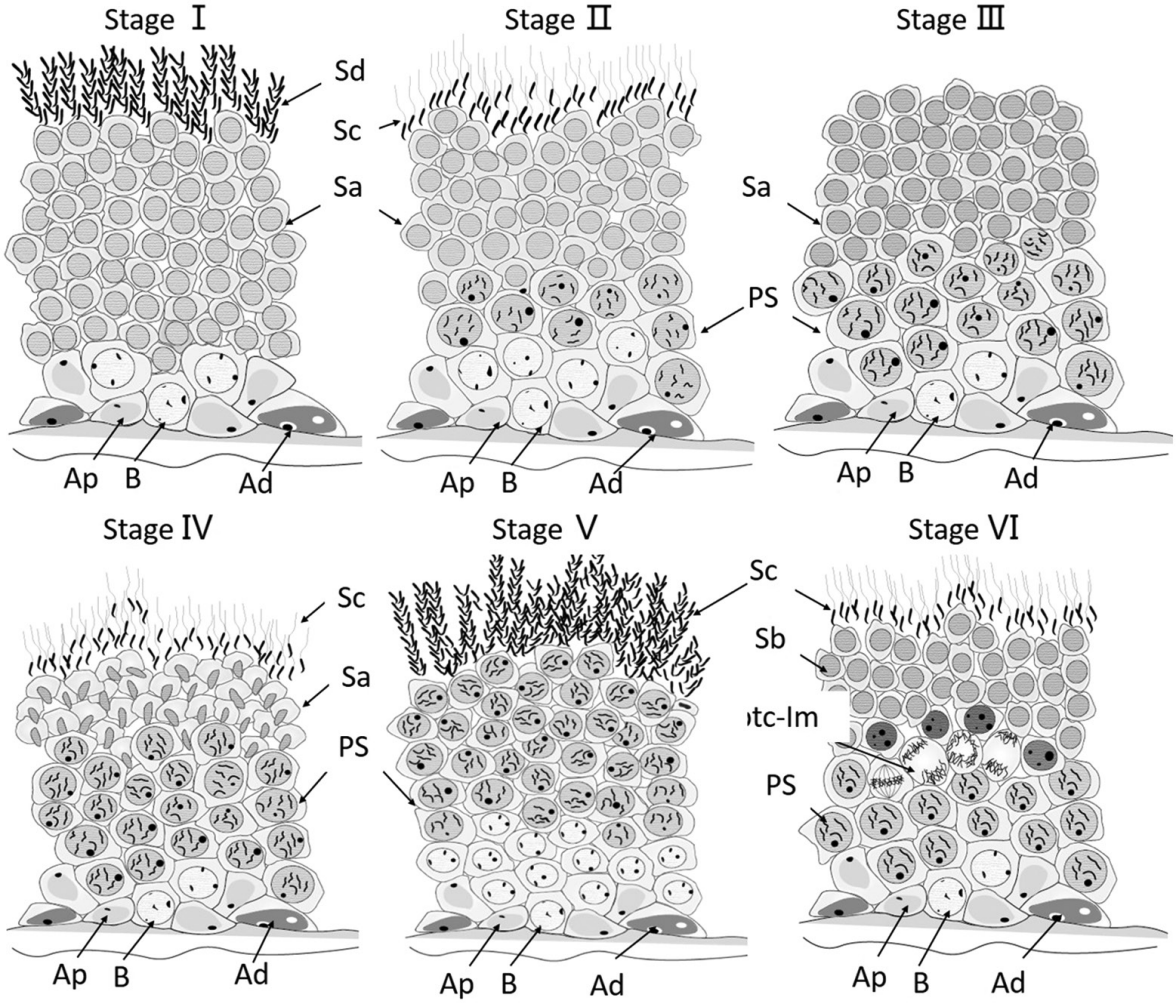
Drawings diagrammatically representing the cellular composition and topography of the six typical cellular associations found repeatedly in Chinese soft-shell turtle seminiferous tubules. These cell associations correspond to the stages of the cycle of the seminiferous epithelium (i.e. stage I–VI). AP, bright spermatogonia; Ad, dark spermatogonia; B, B spermatogonia; PS, primary spermatocytes; STC-Im, spermatocytes in division; Rb, residue; Sa, Sb, sperm cell types of different generations; Sc, Sd, sperm of different generations.

**Figure 4:**
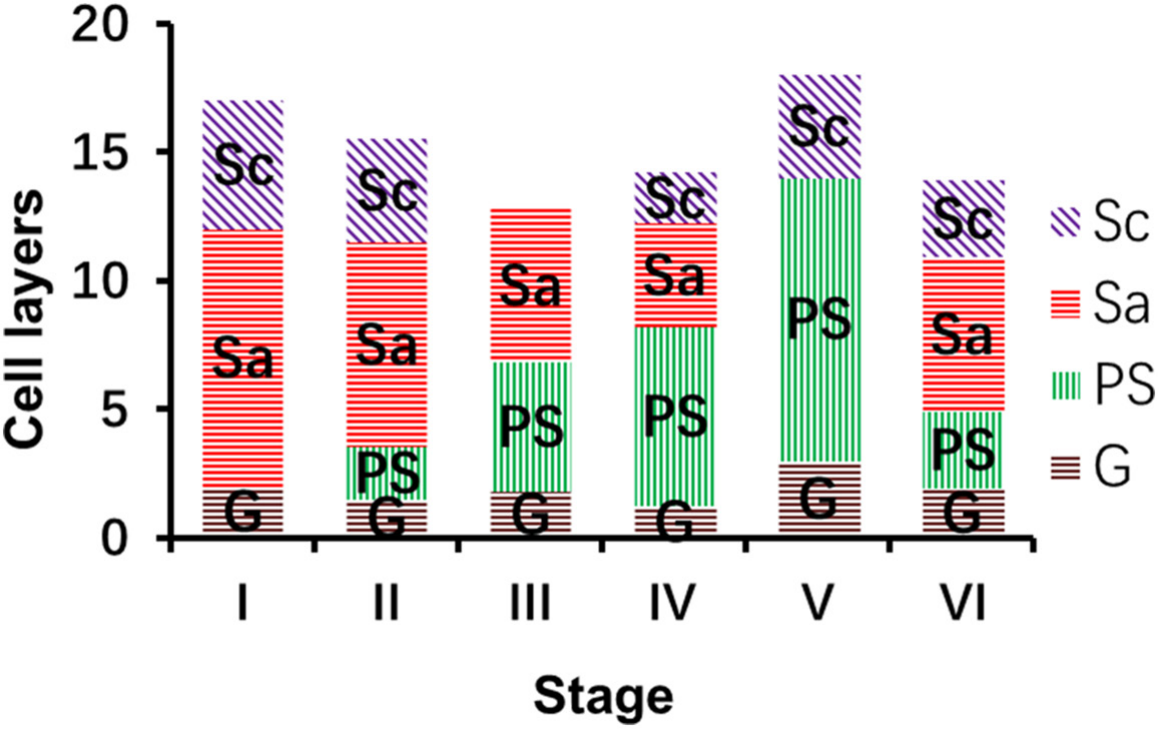
Histogram of the six typical cell combinations of the process of sperm cell development. G, spermatogonia; PS, primary spermatocytes; Sa, Sb, sperm cell types of different generations; Sc, Sd, sperm of different generations.

The photograph of a tubule shows typical stages of the cycles labeled I, II, III, IV, V, and VI, respectively (Fig. 1). The exact composition of the cellular associations was ascertained by examining the tubular histology in serial sections. The descriptions were derived by analyzing H&E-stained sections, and the most characteristic feature of the cell association is described first (Figs. 2, 3 and 4). Each typical cellular association was considered a stage of the cycle of the seminiferous epithelium and is labeled with a Roman numeral.

Stage I of the cycle showed newly formed spermatids (Sa) and the nucleus was round. The sperm (Sd) were developed by the sperm cells (Sb) from the previous stage. The sperm belonging to the transitional stage at this time were not mature. The most obvious feature at this stage was the absence of spermatocytes.

Stage II of the cycle showed generations of young spermatocytes (PS) and fewer layers. The spermatids (Sa) continued to develop, and some of them developed into maturing spermatids (Sc) in advance. The latter were now lining the lumen of the tubule waiting for discharge.

Stage III of the cycle showed that the number of spermatocytes had increased. The mature spermatozoa in the previous stage were discharged, leaving some spermatids (Sa) to continue development.

Stage IV of the cycle showed the generation of spermatids (Sa) with initial signs of elongation of their nuclei. During this time, the maturing spermatids (Sc) developed from a portion of the spermatids (Sa). The older generation of spermatocytes continued to increase, whereas those of the young generation usually just entered prophase of meiosis.

Stage V of the cycle showed that the spermatids (Sa) had developed into a large number of spike-shaped spermatids (Sc). Meanwhile, the number of spermatocytes (PS) reached a maximum.

Stage VI of the cycle showed that the groups of spermatocytes (PS) were undergoing meiotic division (Sptc-Im). A new generation of spermatids (Sb) present at this stage was also indicated. It derived from primary spermatocytes through meiosis.

The six cellular associations or stages of the cycle presented in the order above would, in time, follow each other in sequence. Following stage VI, stage I recurs, and a new cycle begins.

### New pattern of sperm cell development – rapid pattern

**Figure 5:**
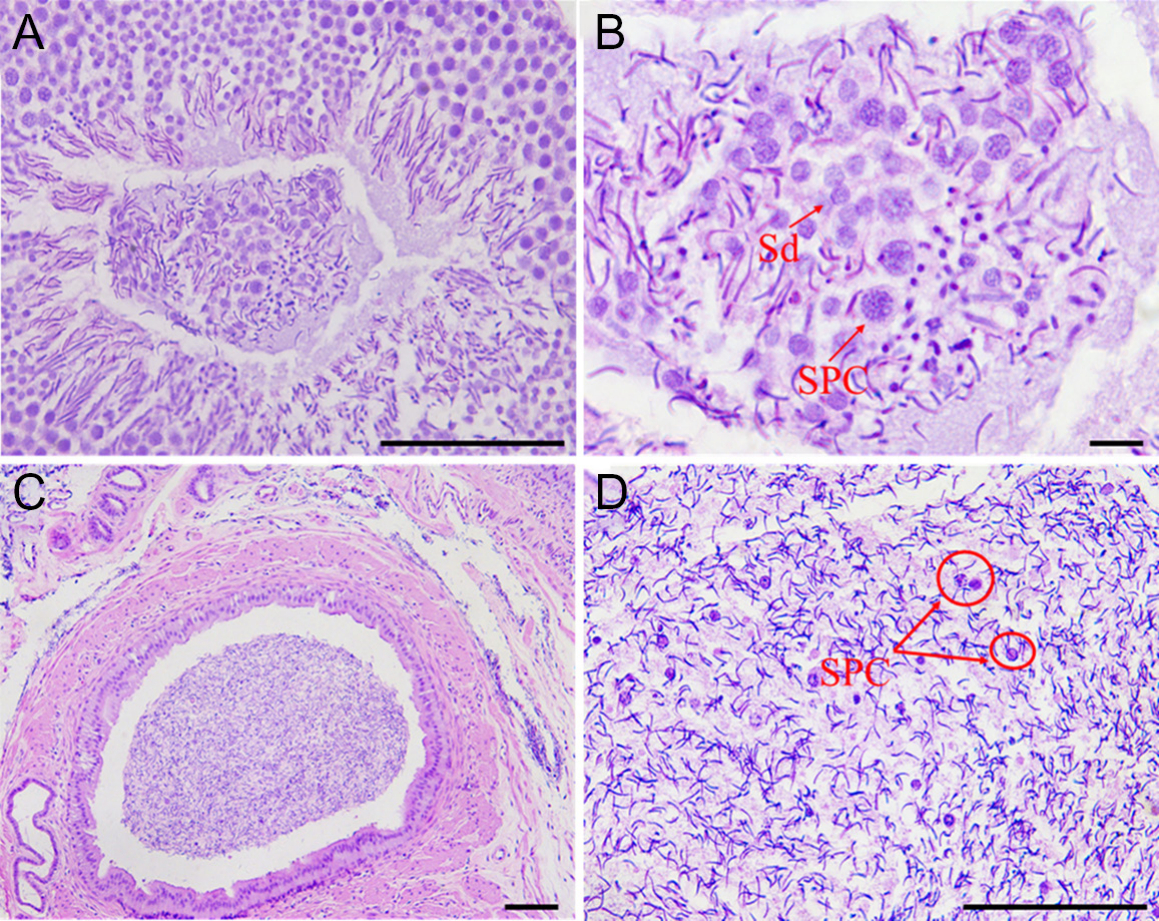
H&E staining of the testicular and epididymal lumen in September samples. SPC, spermatocyte; Sd, spermatid; the scale bar of A, C and D: 100 μm; the scale bar of B: 10 μm.

The new pattern of sperm cell development was ascertained by examining the tubular histology in serial sections. Figure 5A shows a large number of round cells in the lumen of the seminiferous tubules, and Fig. 5B shows the effect at 1000 times magnification. There were not only spermatids but also spermatocytes in the lumen. The epididymal head was examined by the tubular histology in serial sections. We found that there were a large number of circular stained substances in the epididymal lumen (Fig. 5C). Once magnified (Fig. 5D), we observed many circular spermatids interspersed.

**Figure 6:**
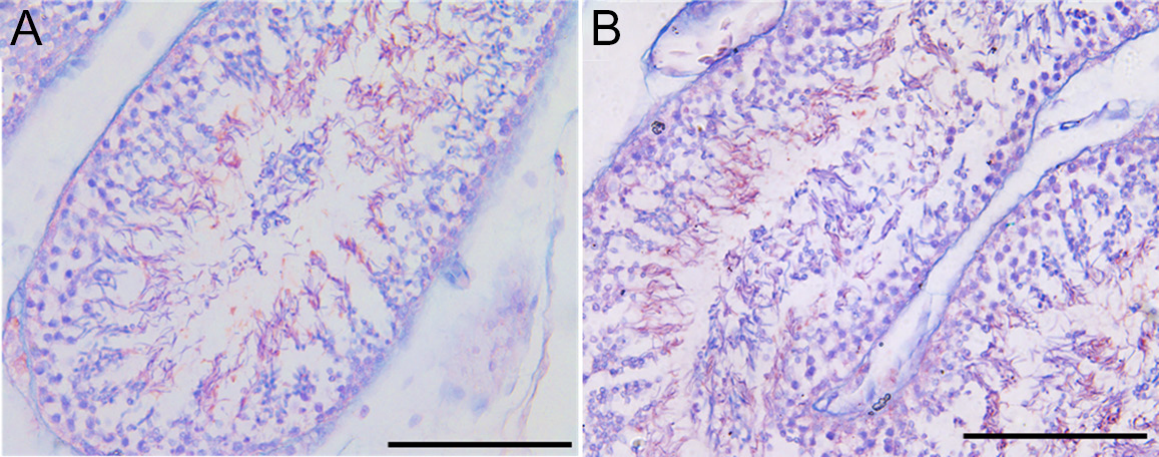
AB-PAS staining indicative of maturity of germ cells during seasonal sperm cell development in Chinese soft-shelled turtle (scale bar: 100 μm).

**Figure 7:**
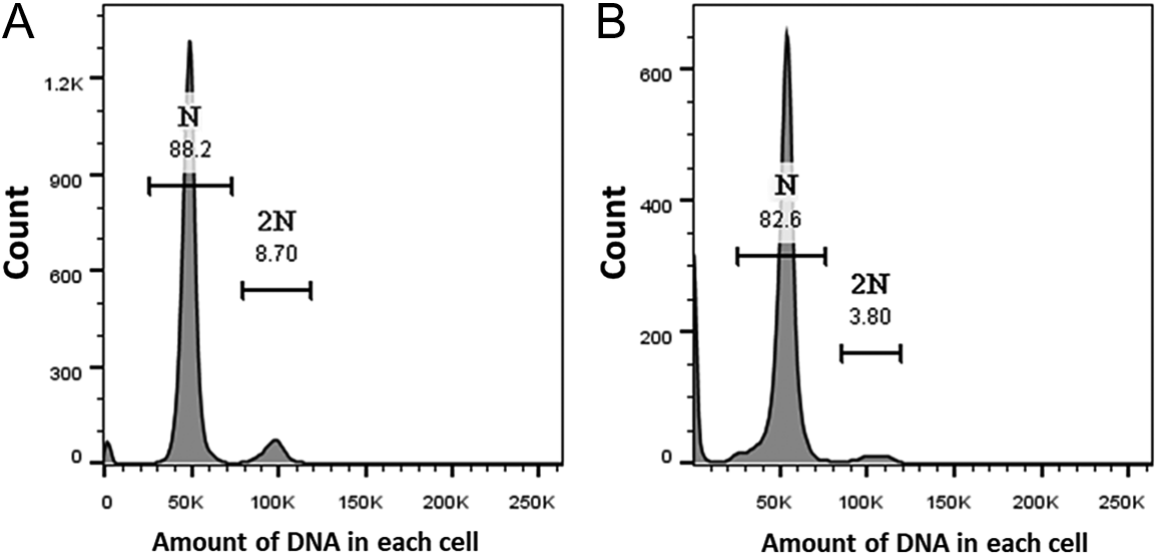
PI staining of spermatozoa in DNA histogram. (A) The semen in the epididymal head in September and (B) the semen in the epididymal head in October.

We observed sperm maturation in testicular cavities using the AB-PAS technique. AB-PAS stains glycogen and neutral mucin purple and acid mucin blue. The shift in color also represents the maturation of sperm. The more mature the sperm, the darker the color. The results showed that the sperm in the lumen were reddish-purple, whereas the round cells in the lumen were not (Fig. 6), which indicates that there were sperm cells or germ cells in the lumen in addition to sperm. To further verify this, we performed flow cytometry on the epididymis. The histograms of the PI-stained samples showed that diploid germ cells existed in the epididymis in September (Fig. 7A) and the number of diploids decreased in October (Fig. 7B). For these 2 months, there were a large number of haploid cells that were sperm.

**Figure 8:**
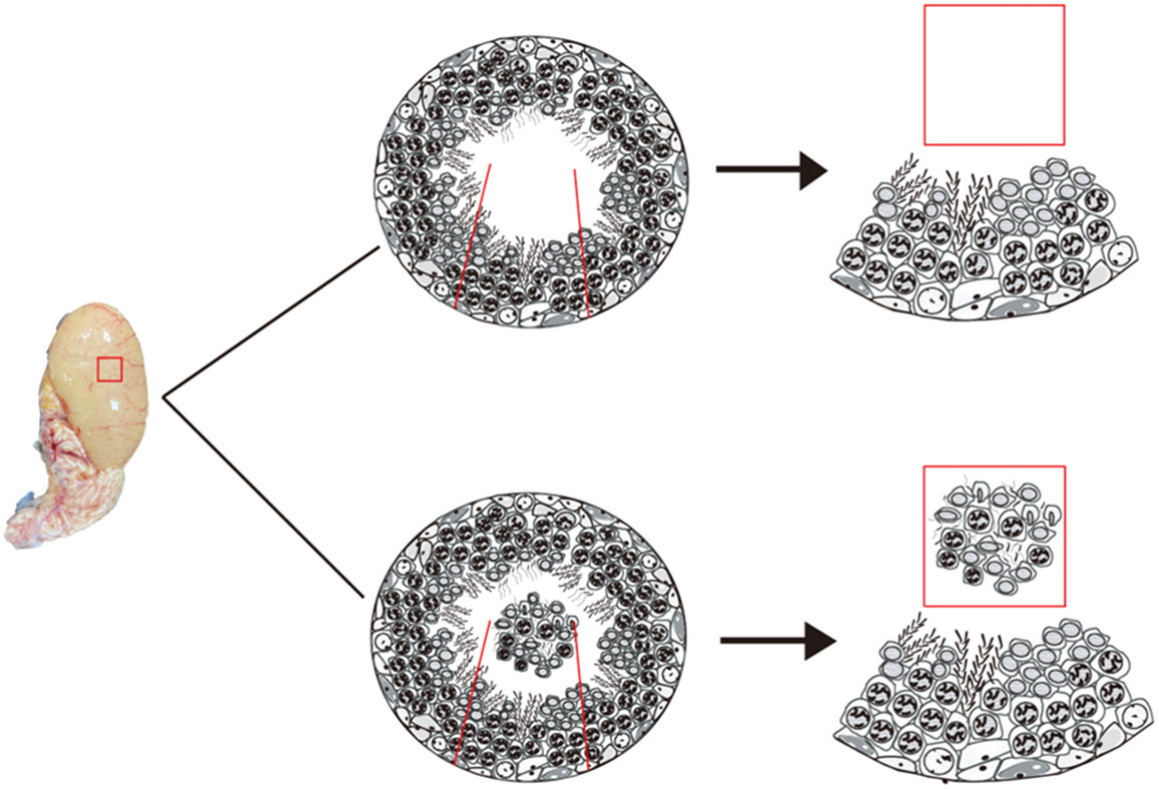
Schematic diagrams of the process of sperm cell development pattern. The upper part represents the normal sperm cell development pattern and the lower part represents the rapid pattern.

To more vividly describe the reproductive cell in the lumen, we prepared an image of the two patterns using a sketch map. Figure 8 shows the two patterns of sperm cell development. The upper part shows the normal pattern. There were no germ cells in the lumen; thus sperm maturation was completed in the seminiferous epithelium. The lower part showed the rapid pattern. In addition to a large number of mature spermatozoa in the lumen, there were germ cells shed from the seminiferous epithelium that were ready to be discharged into the epididymis, where they further develop and mature.

## Discussion

In this study, the six cellular associations described here may be regarded as stages of the cycle. The progress over time occurs in a fixed sequence (from stage I to stage VI) and then repeats time and time again. Based on this characterization, the stages are distinguished mainly by the spermatocytes, which are somewhat different in the way that divides the stages of human seminiferous epithelium described by Clermont (Clermont 1963). The division of human seminiferous epithelium is based on the specific types of spermatocytes and spermatogonia, but it is not enough to draw conclusions by H&E staining only. The determination of germ cell types is very inaccurate and subjective; thus, some studies also use high-resolution light microscopy (HRLM) and transmission electron microscopy to provide high-resolution features for the morphology of human germ cells to accurately divided them into six well-defined stages (Nihi *et al.* 2017).

Currently, the whole germ cells of reptiles are mainly characterized based on the development of the acrosome system of germ cells that divide the reproductive cycle of freshwater turtles into 10 stages (Sousa *et al.* 2014). In the Chinese soft-shelled turtle, some studies have categorized the sperm cell differentiation process into five stages based on changes in sperm nucleus morphology; however, there is little information regarding the high-resolution morphology of germ cells. Most studies observed the structural changes of the sperm nucleus and mitochondrial organelles in mature sperm by transmission electron microscopy, in which they appeared as a manchette structure (Gribbins *et al.* 2003, Chen *et al.* 2020*b*). This change indicates that the structure is associated with the development of the sperm nucleus. This was also observed during the process of sperm cell development of Rhea American albisceus (Sprando & Russell 1988).

An efficient seminiferous epithelial cycle is the key to improving the efficiency of sperm cell development (Fernandes Araujo Chaves *et al.* 2020). In this study, H&E staining was used to identify the morphological changes in germ cells during development. By comparing the process of sperm cell development patterns in the testis, we concluded that there are two different patterns. The first is the normal pattern, in which the process of sperm cell development and metamorphosis is completed in the seminiferous epithelium and then discharged into the epididymis. The second is the rapid pattern, in which the process of sperm cell development and deformation is completed in the lumen of the seminiferous tubule and epididymis at the same time. Therefore, sperm can be discharged into the epididymis before maturation in rapid pattern and further develop and mature; thus, more can be produced in the seminiferous epithelium. Different sperm cells can develop synchronously or less than the former pattern, which greatly improves the proportion of sperm, meets the needs of seasonal reproduction, and improves the probability of successful fertilization.

Under normal circumstances, most sperm mature in the testis. The progenitor cells (spermatogonia) mitotically divide, undergo meiosis, undergo deformation within the seminiferous epithelium of the testis, and then differentiate into a large number of spermatozoa. Finally, they are released into the lumen of the testis. Therefore, the traditional view is that the sperm is the main component of development within the testis. The epididymis is the primary site of sperm storage and maturation. In the rapid pattern, some spermatozoa are transferred to the epididymis for hibernation before they mature, and the development of mature spermatozoa is completed in the epididymis until the next mating season. In addition to mature sperm, there are a large number of undeveloped and some round sperm cells and spermatocytes with obvious heterogeneity in the epididymis, which was already verified in the present study as well as in *Alligator Snapping Turtle* (Trauth & Gribbins 2018). In *Phrynopsgeoffroanus*, the physiological characteristics of sperm storage in the epididymis are similar to those produced during the peak period of the process of sperm cell development, which further indicates that the epididymis is not only a storage organ but also involved in sperm maturation or activation. This provides a theoretical basis for the emergence of the rapid pattern, but whether this pattern only appears at the peak of reproduction needs to be further explored. In the present study, the classification of the cellular features into six stages may serve as a system of landmarks for more detailed histophysiological and histopathological studies on the process of sperm cell development. Moreover, there is a certain correlation between the rapid pattern and the sperm cell development cycle. The difference between the process of sperm cell development patterns is based on a strategy to adapt to seasonal breeding. This allows for heterogeneous and immature spermatozoa to be released from the testis in advance, enabling the epididymis to accumulate a large number of spermatozoa in a short time period to prepare for the next breeding season. Thus, this established model can, to a certain extent, explore the best hatchability and fecundity of sperms. Additionally, according to the characteristics of the process of sperm cell development and maturation, it develops the most suitable diluent or culture medium for *in vitro* preservation of Chinese soft-shelled turtle sperm, expanding the use range of semen. This physiological and ecological phenomenon is a strategic adaptation for animals in response to the internal and external environments that have arisen over the long history of evolution. It is significant for studying the seasonal reproduction of turtles, particularly the rapid production of sperm, which promotes the development of aquaculture.

## Declaration of interest

The authors declare that the research was conducted in the absence of any commercial or financial relationships that could be defined as a potential conflict of interest.

## Funding

This work was supported by the Zhejiang Province New Aquatic Variety Breeding Project (2021C02069-8-3) and Zhejiang Province Public Welfare Technology Application Research Project (LGN21C190013, LGN22C190022) and Major technology research and development projects in Ningbo (2021Z009).

## Author contribution statement

WW conceived the study. YYF handled animals and conducted experiments. YYF, WJH, LRL, YSJ, and QGY conducted result analysis. WW, YYF, and PYD participated in the discussion of the results. YYF wrote the manuscript. WW revised and edited the manuscript. All authors contributed to the finalized manuscript and read, and approved the final version.
